# Measles and rubella serosurvey identifies rubella immunity gap in young adults of childbearing age in Zambia: The added value of nesting a serological survey within a post-campaign coverage evaluation survey

**DOI:** 10.1016/j.vaccine.2019.02.037

**Published:** 2019-04-17

**Authors:** Kyla Hayford, Simon Mutembo, Andrea Carcelen, Hellen K. Matakala, Passwell Munachoonga, Amy Winter, Jane W. Wanyiri, Kelly Searle, Francis D. Mwansa, Angels Mwiche, Caroline Phiri, Chris Book, Philip E. Thuma, William J. Moss

**Affiliations:** a Department of International Health, Johns Hopkins Bloomberg School of Public Health, Baltimore, MD, USA; b Ministry of Health, Government of the Republic of Zambia, Lusaka, Zambia; c Department of Epidemiology and Biostatistics, University of Georgia, College of Public Health, Athens, GA, USA; d Macha Research Trust, Choma, Zambia; e Department of Epidemiology, Johns Hopkins Bloomberg School of Public Health, Baltimore, MD, USA

**Keywords:** Serology, Serological survey, Immunization coverage, Vaccination, Measles, Rubella, Dried blood spots, Surveillance, IgG antibody

## Abstract

**Background:**

Serological surveys can potentially complement vaccine coverage surveys, such as post-vaccination campaign coverage evaluation surveys (PCES), by providing direct information on population immunity within and outside the target age range of the mass vaccination campaign. We estimate age-specific population immunity to measles and rubella viruses in Southern Province, Zambia, and assess the value of adding serological data to vaccination coverage estimates by nesting a serological survey within a PCES.

**Methods:**

Dried blood spots (DBS) from fingerprick blood were collected from all individuals ages nine months or older in households participating in the PCES and tested for measles and rubella virus-specific immunoglobulin G (IgG) by enzyme immunoassay (Siemens Enzygnost, Marburg, Germany).

**Results:**

Overall seroprevalence was 95.5% (95% CI: 92.8, 97.2) for measles virus-specific IgG and 97.7% (95% CI: 96.0, 98.7) for rubella virus-specific IgG. Rubella seroprevalence was 98.4% (95% CI: 95.9, 99.4) among children eligible for the MR vaccination campaign, significantly higher than the reported measles-rubella (MR) vaccination campaign coverage of 89.8% (p = 0.003), and higher than the 91.3% rubella seroprevalence for adolescents and adults 16–30 years of age (p = 0.049).

**Conclusion:**

Seroprevalence to measles and rubella viruses in children younger than 16 years of age was significantly higher than expected from vaccination coverage estimates, likely reflecting exposure to wild-type viruses and underreporting of vaccination. The serosurvey revealed rubella immunity gaps among women 16–30 years of age, precisely the age group in which protection from rubella is most important to prevent congenital rubella syndrome. Nesting serological surveys within existing surveys can leverage resources and infrastructure while providing complementary information important to immunization programs.

## Highlights

• We nested a measles and rubella serological survey in a vaccination coverage survey.
• Measles and rubella immunity was significantly higher than expected by vaccination.
• Study revealed immunity gap in young adults and risk of congenital rubella syndrome.
• Adding serology to a survey leveraged resources and provided complementary information.

## 1 Introduction

Mass vaccination campaigns for measles and rubella are designed to rapidly increase population immunity [1]. Measuring the success of mass vaccination campaigns is critical as these activities are costly and resource intensive [2]. Because vaccine doses from mass campaigns are not consistently recorded on the child’s home-based records (vaccination card), multi-purpose surveys such as the Demographic and Health Surveys or Multiple Indicator Cluster Surveys may underestimate measles and rubella vaccination coverage [3], [4], [5]. Single purpose, probability-based household surveys, such as post-campaign coverage evaluation surveys (PCES), can provide accurate estimates of vaccination coverage within months of the campaign but require expertise in sampling and statistics [6], [7]. Ideally, PCES would be based on accurate vaccination histories derived from home-based vaccination records, but the rapid survey design often results in non-sampling error due to low card retention and reliance on parental recall [4], [8].

Even if probability-based cluster surveys provide accurate vaccine coverage estimates, such surveys do not provide direct measures of population immunity. Coverage evaluation surveys target children eligible for vaccination but population immunity profiles outside the target age group are valuable to assess outbreak risk. Susceptibility to measles is increasingly seen in adolescents and adults and rubella immunity is most important in women of child bearing age [9]. Despite attempts to model population immunity profiles from vaccination coverage and disease incidence, these inferences often deviate from true population immunity in transmission settings where both vaccination and wild-type virus infection occur [12].

Serological surveys can potentially complement PCES by providing direct information on population immunity within and outside the target age range of the mass vaccination campaign [10], [11], [12]. In addition, serological surveys can leverage the PCES sampling frame and infrastructure to be more efficient than stand-alone serosurveys [13], [14]. This study evaluated age-specific population immunity to measles and rubella viruses in Southern Province, Zambia, by conducting a serological survey nested within a PCES to assess the value of adding serological data to vaccination coverage estimates.

## 2 Methods

### 2.1 Post-campaign coverage evaluation survey

A national catch-up measles and rubella (MR) vaccination campaign was conducted in Zambia from 19th to 24th September 2016 targeting all children 9 months to younger than 15 years of age. In practice, however, children 15 years old were included in the vaccination campaign and therefore this analysis considered children 9 months to younger than 16 years of age as eligible for the MR campaign. This campaign was the first time rubella-containing vaccine was administered through the public sector in Zambia. Vaccination cards were provided during the campaign.

From 21st November to 3rd December 2016, a national PCES was conducted to assess vaccination coverage achieved by the campaign and routine immunization program. We partnered with the PCES team to conduct a serological survey concurrent with the PCES in Southern Province, Zambia, to estimate age-specific population immunity to measles and rubella viruses. The serosurvey was approved by Institutional Review Boards at Macha Research Trust (#E2016.04) and the Johns Hopkins Bloomberg School of Public Health (#00007447). Regulatory approval for the PCES and serosurvey was granted by the National Health Research Authority in Zambia (MH/101/23/10/1).

### 2.2 Sampling strategy

The serosurvey followed the PCES sampling strategy, a two-stage cluster survey in which the primary sampling unit (PSU) is a ward selected from the 2010 census with probability of selection proportional to estimated size. There were 192 wards in Southern Province. For the second stage, the sampling frame was constructed by mapping all households in the PSU and a systematic random sample of 12 households with at least one eligible child was selected, stratified by whether or not all children younger than 16 years of age in the household participated in the vaccination campaign. The latter group was oversampled and weighted accordingly to achieve sufficient response rates for PCES questions on reasons for not participating in the vaccination campaign. Due to logistical constraints, four of seven PCES teams were randomly selected to nest the serosurvey and therefore 15 of the 26 PSUs in Southern Province were included in the serosurvey. Whereas the PCES included children 9 months to younger than 16 years of age, all members of the household 9 months of age and older at the time of the MR campaign were eligible to participate in the serosurvey. Because we worked within the PCES procedures for household selection, no revisits were conducted.

The measles and rubella serosurvey was designed to estimate seroprevalence within ±7% for each antigen and for each age group (9 months to younger than 5 years; 5 years to younger than 16 years; and 16 years and older) in Southern Province, Zambia. Sample size was calculated using the World Health Organization (WHO) Cluster Survey Manual, which estimates the minimum number of complete enrollments required based on assumptions about the seroprevalence and survey design: Minimum number of complete interviews = (number of strata) × (effective sample size) × (design effect) [15]. We assumed 81% population immunity and 1.46 design effect based on a previous measles and rubella serosurvey among HIV-infected children [16]. Because the serosurvey was nested in the PCES, calculations were constrained to the number of households selected for the PCES and aimed to enroll the minimum number of children from the youngest and smallest age group. Because more older children and adults were available than required in the selected households and seroprevalence was higher than assumed, observed seroprevalence estimates were more precise than predicted.

### 2.3 Measles and rubella serosurvey

The serosurvey was conducted as a research study in partnership with the programmatic PCES. The PCES team administered a vaccination history questionnaire adapted from the WHO Cluster Survey Manual [15]. The serosurvey team recorded the results of the PCES questionnaire and asked additional questions to participants outside the PCES age range. If campaign or routine vaccination cards were available, dates or evidence of vaccination were recorded. If vaccination information was missing on the card or a card was not available, vaccination status was based on caregiver recall.

### 2.4 Sample collection and processing

A fingerprick blood sample was collected using a retractable lancet and a maximum of five spots were spotted on Whatman 903 Protein Saver dried blood spot (DBS) cards. In a subset of clusters where specimens could be transferred within 24 hours to the laboratory at Macha Research Trust, 200–300 μL of blood was collected by fingerprick in a serum separator BD Microtainer® tube. DBS cards were dried for eight hours, placed in plastic storage bags with a desiccant, and kept at room temperature for 1–3 days until transfer to the laboratory for long-term storage at −20 °C. Liquid blood was stored at 2–8 °C in the field, centrifuged and stored at −20 °C within 24 hours of collection. Detailed methods are described in Appendix A.

### 2.5 Measles and rubella enzyme immunoassays

Serum eluted from DBS were tested for immunoglobulin G (IgG) antibodies to measles and rubella viruses with indirect enzyme immunoassays (EIA, Enzygnost; Siemens, Munich, Germany) at Macha Research Trust. Specimens were classified as positive (>0.2 corrected optical density (cOD)), equivocal (0.1–0.2 cOD), or negative (<0.1 cOD). For samples with cOD > 0.1, measles (mIU/mL) and rubella (IU/mL) antibody concentrations were calculated according to the manufacturer’s protocol. Equivocal results were re-tested and, if equivocal again, were categorized as positive for analyses.

Three adjustments were made to the DBS results to account for volume, spot size and diagnostic accuracy of DBS compared to serum, described in Appendix B. Briefly, antibody results were adjusted using linear regression from a known DBS panel for samples with eluate volumes less than 50 μL and for DBS less than 12 mm in circumference [16]. Results were also adjusted for the observed diagnostic accuracy of capillary whole blood DBS compared to capillary serum. Paired serum and DBS specimens from 203 individuals in the study were tested on the same plates to generate an adjustment factor for DBS. IgG antibody concentrations and qualitative classifications (positive, equivocal, negative) were calculated using the adjusted cOD values. Secondary analyses were conducted with unadjusted results.

### 2.6 Statistical analysis

Results were weighted to account for the inverse probability of selection at each stage, non-response and post-stratification adjustments. Weighted analyses are presented with 95% logit confidence intervals (CI). Local polynomial smoothing was used to model age-specific seroprevalence. Categorical variables and age-specific vaccination coverage and seroprevalence estimates were compared using Rao-Scott chi-square tests and continuous variables with non-normal distributions were compared using Wilcoxon rank sum tests. All analyses accounted for survey weights. We aimed to extrapolate results from the serosurvey clusters to the entire province by building prediction models. Models considered individual and household level predictors that were chosen *a priori* given known characteristics of serostatus (i.e., age, vaccination status) or selected via random forest analyses (Appendix C, Figs. S2–S4).

## 3 Results

**Fig. 1:**
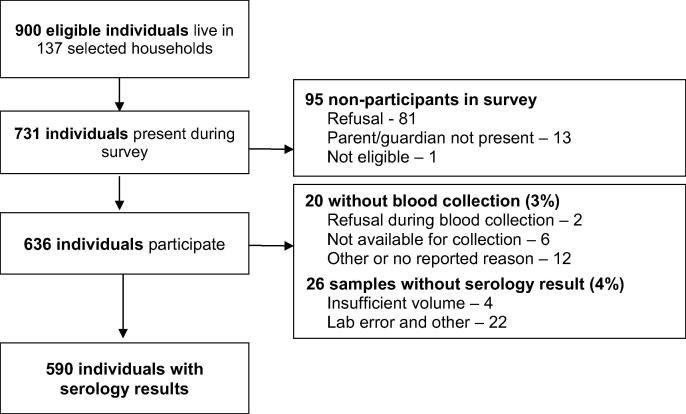
CONSORT enrollment flow diagram.

Data collection occurred over 13 days in 15 survey clusters in Southern Province, Zambia during November and December 2016. Three clusters were in urban areas and 12 clusters in rural areas (Supplementary material, Fig. S1). One PCES cluster was replaced using non-probability sampling and therefore was not included in the serosurvey. A total of 900 individuals resided in the 149 households selected for the survey, of whom 81% (731 individuals) were present at the time of the survey (Fig. 1). Of those present, 87% (636) agreed to participate in the serosurvey. Eleven percent (81) refused, 2% (13) did not have a parent or guardian present, and one child was ineligible. Blood was collected from 97% (616) of participants. There was no difference in blood collection refusal by age group (p = 0.15) or sex (p = 0.81). A total of 590 specimens were tested for measles and rubella IgG, representing 65% of all eligible residents, 81% of all residents present at the time of the survey, 93% of those who agreed to participate in the serosurvey, and 96% of those from whom a blood sample was collected. 26 specimens were not tested due to insufficient volume or laboratory error (Fig. 1).

Sixty two percent of specimens were collected from children 9 months to younger than 16 years of age at the time of the MR campaign and were therefore eligible for MR vaccination. Among children, an equal proportion of girls and boys were enrolled. Outside the campaign age range, 68% were female. Sixty percent of household heads were farmers and 10% had completed secondary school. Seventy one percent of participating households reported traveling at least 30 min to the nearest vaccination clinic (Table 1).

**Table 1:** Participant and household characteristics**.**

**Participant characteristics**^1^	
Number of participants with blood sample	590
Female – 9 months to younger than 16 years (%)	48.9
Female – 16 years and older (%)	67.6
Age, median [IQR]	11.7 [6.3, 28.2]
9 months to <5 years (%)	19.7
5–<16 years (%)	42.2
16 years and older (%)	38.1

**Household characteristics**^1^, ^2^	
Number of households	143
Household size, median [IQR]	7 [5, 9]
Participants per household, median [IQR]	4 [3, 6]
Maternal age (%)	
<30 years	12.3
30–39 years	22.2
40–49 years	28.8
50 years and older	28.9
Father’s occupation (%)	
Farm laborer	58.5
Business/self-employed	15.6
Paid employment	13.3
Other	9.2
Reported travel time to vaccination clinic (%)	
<30 min	34.2
30–59 min	31.4
60 min or more	32.0

1 Survey weighted proportions presented.

2 Household level characteristics percentages may not add up to 100% because some data were not available for households, not included in PCES or due to missing data.

### 3.1 Measles and rubella vaccination coverage

**Fig. 2:**
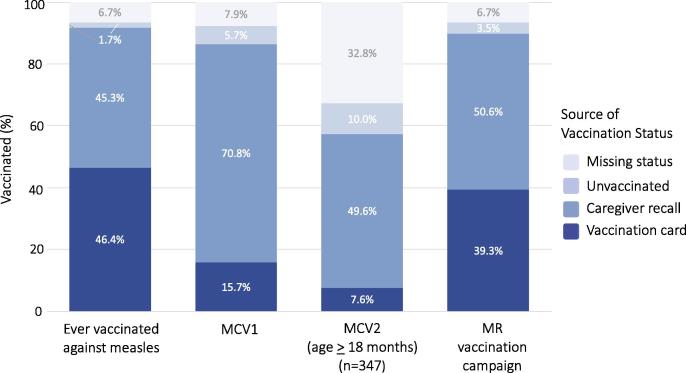
Measles and rubella vaccination coverage among children younger than 16 years of age (n = 368). Notes: MCV1: First dose of measles-containing vaccine from routine vaccination system. MCV2: Second dose of measles-containing vaccine from routine vaccination system among children ages 18 months or older at time of survey. MR: measles-rubella. Receipt of MR vaccination campaign dose recorded on campaign immunization cards. Routine MCV1 and MCV2 recorded on separate home-based immunization card. Children ever vaccinated for measles had evidence of MCV1, MCV2, or MR vaccination campaign dose. Some children were missing data on vaccination status: 1.1% ever vaccinated for measles and MR vaccination campaign, 2.7% for MCV1 and 11.0% for MCV2. Sum of coverage by vaccination card and caregiver recall may vary slightly from overall coverage reported in manuscript due to weighting.

An estimated 89.9% (95% confidence interval (CI): 85.9, 92.8) of eligible children ages 9 months to younger than 16 years received MR vaccine during the campaign. Only 39.3% of caregivers provided a campaign vaccination card, and 50.6% reported the child received the campaign vaccine but did not show a card (Fig. 2). There was no difference in campaign card retention between older and younger children (Appendix D, Table S1). Because the MR campaign was the first opportunity to receive rubella vaccination in the public sector, campaign coverage reflected rubella vaccination coverage in this age group.

**Fig. 3:**
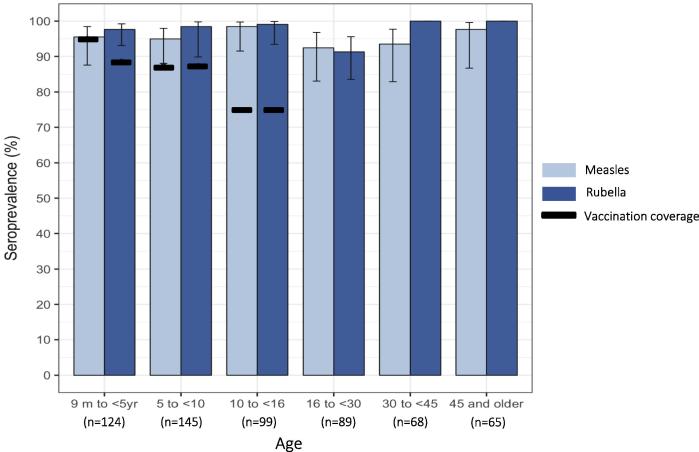
Age-specific measles and rubella IgG seroprevalence and vaccination coverage in Southern Province, Zambia (n = 590). Notes: Light and dark blue bars and 95% logit confidence intervals represent measles and rubella IgG seroprevalence, respectively. 95% confidence intervals were not calculated for strata with 100% seroprevalence. Equivocal results classified as positive. Black line represents vaccination coverage among children aged nine months to younger than 16 years based on evidence from the card or parental recall. Rubella vaccination coverage based on receipt of the measles-rubella vaccine from the campaign. Measles vaccination coverage based on receipt of any measles-containing vaccine through the routine immunization system or mass vaccination campaign. (For interpretation of the references to colour in this figure legend, the reader is referred to the web version of this article.)

Children had up to two additional opportunities for measles vaccination through the routine immunization system. 86.1% (95% CI: 81.8, 89.5) of children reportedly received MCV1 and 57.6% (95% CI: 48.7, 66.0) of children who were at least 18 months of age received MCV2 based on their routine vaccination card or parental recall (Fig. 2, Fig. 3). Combined with campaign vaccines, 91.7% (95% CI: 88.0, 94.3) of children received at least one dose of MCV from a campaign or the routine immunization system. The campaign increased measles vaccination coverage by 13.4% among children less than five years of age.

Measles vaccination history was based largely on caregiver recall, especially among older children (Table S1). Only 57% (95% CI: 48.1, 65.8) of caregivers showed a routine vaccination card, and 15.8% of MCV1, 7.6% of MCV2 and 39.4% of MR campaign doses were verified by a vaccination card. Vaccination status for MCV1 or the MR campaign was missing for 7.9% and 6.7% of children, respectively (Fig. 2). Unknown vaccination status was significantly higher among older children for MCV1 (10.3% among children 5 to younger than 16 years of age and 2.7% among children 9 months to younger than 5 years; p = 0.03, Table S1) and the MR campaign (9.3% and 1.1%, p = 0.01).

### 3.2 Measles seroprevalence

**Fig. 4:**
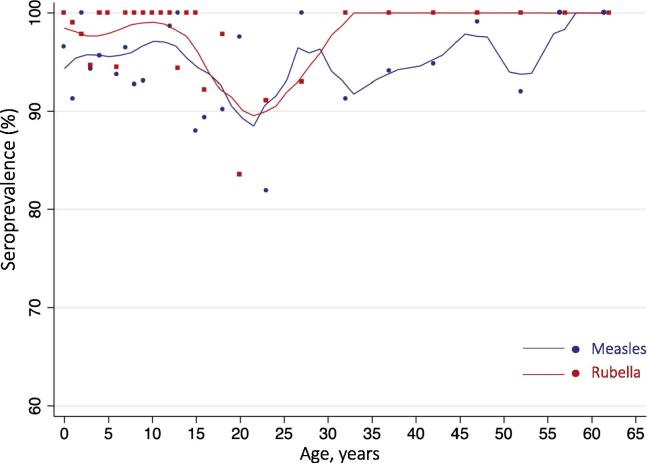
Age-specific measles and rubella seroprevalence among serosurvey participants in Southern Province, Zambia (n = 590). Notes: Age-specific measles and rubella IgG seroprevalence in blue and red, respectively. Dots (•) represent seroprevalence point estimates for each age group using age bins with approximately equal sample size, one year intervals for ages 9 months to 15 years, two year intervals for ages 16–19 years, five year intervals for ages 20–65 years. Lines are smoothed seroprevalence estimates using kernel-weighted local polynomial regression. Equivocal results were classified as positive. (For interpretation of the references to colour in this figure legend, the reader is referred to the web version of this article.)

The proportion seropositive for measles virus-specific IgG (cOD > 0.2) was 95.5% (95% CI: 92.8, 97.2) across all age groups (Table 2). A small proportion (4%) of samples had equivocal results (cOD 0.1–0.2) on both the initial and second test and were classified as positive. Measles seroprevalence among children eligible for the campaign was 96.1% (95% CI: 92.4, 98.1), which was not significantly different from the seroprevalence above the target age range (94.5%, 95% CI: 89.2, 97.2, p = 0.48) (Fig. 3). Seroprevalence was lower for individuals 16 to less 30 years of age but no age group was lower than 80% (Fig. 4). At 30 years of age and older, measles seroprevalence was over 90% and increased with age.

**Table 2:** Measles and rubella IgG seroprevalence by age and immunization status.

	Measles seropositive % (95% CI)^1^	Measles seronegative % (95% CI)	Rubella seropositive % (95% CI)	Rubella seronegative % (95% CI)
***9 months to <5 years (n = 124)***	95.5 (87.6 98.5)	4.5 (1.5, 12.4)	97.6 (93.1, 99.2)	2.4 (0.8, 6.9)
**MR campaign (ever vaccinated for rubella)**				
Not received	8.2 (3.9, 16.4)	0^*^	7.3 (3.3, 15.2)	30.0 (0.9, 95.4)
Received	90.7 (82.4, 95.3)	100^*^	91.6 (83.7, 95.9)	70.0 (4.6, 99.1)
*Verbal recall*	*40.6 (29.5, 52.9)*	*16.5 (9.1, 81.0)*	*40.5 (29.4, 52.6)*	0^*^
*Card verified*	*50.1 (38.7, 61.4)*	*83.5 (19.0, 99.1)*	*51.1 (39.8, 62.3)*	*70.0 (4.6, 99.1)*
Unknown	1.1 (0.2, 5.8)	0^*^	1.1 (0.2, 5.7)	0^*^

**Ever vaccinated for measles**				
Not received	2.2 (0.6, 8.2)	0^*^	–	–
Received	96.6 (90.8, 98.8)	100^*^	–	–
*Verbal recall*	*40.0 (29.6, 51.5)*	*83.5 (19.0, 99.1)*	–	–
*Card verified*	*56.6 (44.9, 67.6)*	*16.5 (0.9, 81.0)*	–	–
Unknown	1.1 (0.2, 5.8)	0^*^	–	–

***5–<16 years (n = 244)***	96.4 (92.4, 98.4)	3.6 (1.6, 7.6)	98.7 (94.5, 99.7)	1.3 (0.3, 5.4)

**MR campaign (ever vaccinated for rubella)**				
Not received	1.4 (0.6, 3.4)	2.4 (0.1, 30.3)	1.1 (0.4, 2.7)	30.2 (0, 100)
Received	89.4 (84.4, 92.8)	86.8 (34.4, 98.8)	89.5 (84.4, 93.1)	69.8 (0, 100)
*Verbal recall*	*51.5 (41.1, 61.7)*	*13.0 (0.8, 73.0)*	*50.7 (40.5, 60.9)*	0^*^
*Card verified*	*37.9 (27.6, 49.4)*	*73.8(24.0, 96.2)*	*38.8 (28.5, 50.1)*	*69.8 (0, 100)*
Unknown	9.2 (5.9, 14.1)	10.8 (0.7, 68.7)	9.4 (6.0, 14.5)	0^*^

**Ever vaccinated for measles**				
Not received	1.4 (0.6, 3.4)	2.4 (0.1, 30.3)	–	–
Received	89.4 (84.4, 92.8)	86.8 (34.4, 98.8)	–	–
*Verbal recall*	*48.1 (38.1, 58.3)*	*13.0 (0.8, 73.0)*	–	–
*Card verified*	*41.3 (31.2, 52.1)*	*73.8 (24.0, 96.2)*	–	–
Unknown	9.2 (5.9, 14.1)	10.8 (0.7, 68.7		
16–<45 years (n = 157)	92.9 (86.9, 96.3)	7.1 (3.7, 13.1)	95.1 (90.4, 97.5)	4.9 (2.5, 9.6)
Female	92.4 (85.9, 96.1)	7.6 (3.9, 14.1)	94.5 (89.5, 97.2)	5.5 (2.8, 10.5)
Male	93.1 (85.4, 97.0)	6.8 (3.0, 14.6)	92.5 (85.8, 96.2)	7.5 (3.8, 14.2)
45+ years (n = 65)	97.7 (87.2, 99.6)	2.3 (0.4, 12.8)	100^*^	0^*^
Female	96.3 (80.8, 99.4)	3.7 (0.6, 19.2)	100^*^	0^*^
Male	100^*^	0^*^	100^*^	0^*^

Asterisk (^*^) indicates confidence intervals were not calculated because estimate is 0, 100 or sample size is too small to estimate.

1 Estimates account for survey weighting. 95% logit confidence intervals.

### 3.3 Rubella seroprevalence

The proportion seropositive for rubella virus-specific IgG (cOD > 0.2) was 97.7% (95% CI: 96.0, 98.7) (Table 2). Three samples tested equivocal (cOD 0.1–0.2) repeatedly and were classified as positive. Lower rubella seroprevalence was most notable among participants 16 to younger than 30 years of age (Fig. 3, Table 2). Rubella seroprevalence among children eligible for the MR campaign was 98.4% (95% CI: 95.9, 99.4), significantly higher than the seroprevalence of 91.3% (95% CI: 83.6, 95.5; p = 0.049) for adolescents and adults 16 to younger than 30 years of age (Fig. 4). When restricting to females only, all women older than 45 years and 98% of girls younger than 16 years of age were rubella seropositive. However, rubella seroprevalence was only 88% (95% CI: 77.4, 94.2) among women 16–30 years of age (Fig. 4). Rubella seroprevalence was significantly lower among women of reproductive age than children eligible for the campaign in unweighted analyses, but a statistically significant difference was not detected in the weighted analysis (p = 0.06). As for measles, rubella seroprevalence was not lower than 80% in any age group. Sensitivity analyses using unadjusted serology results for measles and rubella did not change inferences. Prediction models were developed to predict measles and rubella serostatus in clusters not selected for the serosurvey and generate seroprevalence estimates for the entire province. Due to the small number of seronegative individuals in this sample, rubella and measles serostatus were not generalizable based on observed covariates and therefore seroprevalence could not be extrapolated to the Southern Province (Appendix C, Tables S2–S3). The observed design effect was 1.56 for measles and 1.10 for rubella seroprevalence.

### 3.4 Comparison of vaccination status and measles and rubella seroprevalence

Measles and rubella specific IgG from the campaign dose were expected to be detectable in immunized children [17]. Among children younger than 5 years of age, rubella seroprevalence was significantly higher than MR campaign vaccination coverage (exact McNemar’s test, p = 0.003) (Fig. 3). At the individual level, 91.6% of the rubella seropositive children had a concordant vaccination history; 40.5% verified with a campaign vaccination card and 51.1% reported by the caregiver (Table 2). 7.3% of rubella seropositive children did not receive a campaign dose and 1.1% were missing vaccination history data. Only 2.4% of young children lacked protective levels of rubella antibodies, limiting statistical power to analyze rubella seronegative children by vaccination status. Similarly, 89.5% (95% CI: 84.4, 93.1) of children 5 to less than 16 years of age had concordant vaccination history, with 38.8% by campaign vaccination card and 50.7% by caregiver recall.

Among children younger than 5 years, 96.6% (95% CI: 90.8, 98.8) of those seropositive for measles reported ever being vaccinated with a measles vaccine, 40% by verbal recall and 57% verified by card. Only 2.2% (95% CI: 0.6, 8.2) of seropositive individuals reported never receiving a measles vaccine and 1.1% (95% CI: 0.2, 5.8) were missing vaccination history data. All measles seronegative individuals reported receiving at least one dose of measles vaccine but only 16% were verified with a vaccination card. Campaign vaccination history and routine vaccination history for children 5–16 years of age were missing for 9.3% (95% CI: 5.9, 14.4) and 10.3% (95% CI: 6.8, 15.5) of participants (Table S1). Among those with missing vaccination data, all children were rubella seropositive and 95.8% (95% CI: 78.2, 99.3) were measles seropositive.

## 4 Discussion

A serological survey of measles and rubella antibodies was successfully conducted in conjunction with a PCES and provided additional information not readily apparent from the vaccination coverage data. First, seroprevalence to measles and rubella viruses was significantly higher than vaccination coverage estimates in children younger than 16 years of age. For rubella, this most likely represents exposure to wild-type rubella virus as the campaign was the first introduction of rubella vaccine through the national immunization program in Zambia. The higher level of population immunity to measles virus likely represents a combination of underreporting of measles vaccination and exposure to wild-type measles virus. The Government of Zambia conducted national measles vaccination campaigns in 2000, 2002/03, 2007, 2010 and 2012 with reportedly high coverage for wide pediatric age ranges. Because vaccinations received during campaigns are not consistently recorded on vaccination cards, measles vaccination coverage may have been underestimated. After a large measles outbreak in 2010 and 2011, Zambia has reported low levels of transmission with fewer than 100 confirmed measles cases per year since 2013 [18]. Regardless of the reason, the high levels of population immunity to measles and rubella viruses at the time of the serosurvey are sufficient to interrupt measles and rubella virus transmission in the absence of pockets of susceptibles. Because of the low number of seronegative individuals, we were not able to assess spatial clustering of susceptible individuals or risk of focal outbreaks, which would increase the critical vaccination coverage necessary to interrupt measles virus transmission.

Second, the serosurvey revealed immunity gaps among young adults not eligible for the campaign, gaps which would not have been identified through the PCES. Specifically, lower rubella seroprevalence (88%) was identified through the serological survey in women 16 to younger than 30 years of age, precisely the age group in which protection from rubella is most important to prevent congenital rubella syndrome [19]. The MR vaccination campaign ensured high levels of rubella seroprevalence in children younger than 16 years of age and cumulative exposure to wild-type virus ensured high seroprevalence among women older than 45 years. However, some older girls and women above the age group targeted in the campaign had not yet been exposed to wild-type rubella virus and remained susceptible. These findings suggest a low but potential risk of rubella in pregnancy for the cohort of women of child bearing age above the age of eligibility for the MR campaign. Surveillance for congenital rubella syndrome in South Africa revealed a potential immunity gap among women 14–30 years of age and a serosurvey in Namibia in 2010 identified the lowest rubella immunity among women 15–19 years of age [20], [21]. High levels of population immunity to rubella need to be maintained through routine immunization services to reduce the risk of rubella virus transmission in this setting. Gavi, the Vaccine Alliance, is supporting 25 low and middle income countries to introduce rubella-containing vaccines by 2020 [22]. Monitoring both vaccination coverage and seroprevalence will be important to monitor the risk of rubella and congenital rubella syndrome risk in this transition period.

Results of serosurveys are also prone to selection and misclassification biases [23]. Although selection of clusters for the PCES was based on probability sampling, not all clusters agreed to participate in the serosurvey, not all eligible household residents were available at the time of the serosurvey, not all residents who were available agreed to or could participate in the serosurvey, and not all of those who agreed to participate had a blood sample with a valid test result. We do not know to what extent non-participation biased our estimates of measles and rubella seroprevalence by age and sex. The enzyme immunoassays lack perfect sensitivity and specificity and, unlike plaque reduction neutralization assays, do not measure functional, protective antibodies. Misclassification of serostatus is most likely among individuals with low but protective antibody levels [24], [25].

Collecting blood on a subsample of participants and extrapolating results to the target population using prediction modeling is a desirable approach to reduce costs and logistical challenges of a serosurvey. Due to the high seroprevalence observed in this study, there was insufficient variation in serostatus to build predictive models and estimate population immunity for Southern Province, Zambia. In post-campaign settings, when seroprevalence is very high, prediction models may be difficult to build (Appendix C).

A serosurvey conducted with a PCES in Southern Province, Zambia provided additional, important information on the high levels of population immunity to measles and rubella viruses following a mass MR vaccination campaign and identified a potentially important rubella immunity gap in adolescent girls and women of child bearing age. Vaccination coverage estimates of the target age group for MR vaccination would not have provided this information, regardless of how accurate and precise. Nesting serological surveys within existing surveys can leverage resources and infrastructure while providing complementary information important to immunization programs.
