# Supplementary material for: Measles and rubella serosurvey identifies rubella immunity gap in young adults of childbearing age in Zambia: The added value of nesting a serological survey within a post-campaign coverage evaluation survey
Source: Vaccine. 2019 Apr 17;37(17):2387–93. doi: 10.1016/j.vaccine.2019.02.037 (PMC6467544; doi:10.1016/j.vaccine.2019.02.037)
Supplement: Supplementary data 1 [file mmc1.docx]

**Supplementary Materials**

**Appendix A. Protocol for processing and testing dried blood spots with Enzygnost enzyme immunoassays for measles and rubella virus IgG**

A fingerprick blood sample was collected using a retractable lancet (BD Microtainer® contact-activated lancet, adult and child size, Becton, Dickinson and Company, Franklin Lakes, NJ) and a maximum of five spots were spotted on Whatman 903 Protein Saver dried blood spot (DBS) cards (GE Healthcare Ltd., New Jersey, USA). In a subset of clusters where specimens could be transferred to the laboratory at Macha Research Trust in Choma District, Southern Province within 24 hours, 200 to 300 μL of blood was collected by fingerprick in a serum separator BD Microtainer® tube (Becton, Dickinson and Company, Franklin Lakes, NJ).

DBS cards were dried and placed in labeled, sealed Whatman storage bags with one Whatman desiccant pack per bag at room temperature for 1-3 days until transfer to the laboratory at Macha Research Trust, where they were stored in air tight containers at -20° C until testing. Liquid blood was stored in a cold box at 2-8° C in the field, transferred to the laboratory at the end of each day, and centrifuged within 24 hours of collection. Sera were stored at -20° C until testing, approximately three months after collection.

Serum eluted from DBS were tested for IgG antibodies to measles and rubella viruses with indirect enzyme immunoassays (EIA, Enzygnost; Siemens, Munich, Germany) at Macha Research Trust. The circumference of each DBS was measured, cut with sterilized scissors, and serum was eluted with 250 μL of buffer as described in protocol 6 in Mercader et al.^1^ Fifty microliters of eluted sample were transferred to precoated 96-well plates and the manufacturer’s protocol was followed to perform the EIA. The optical density (OD) was read at 450 nm using a BioTek ELx800 microplate reader (BioTek Instruments, Winooski, VT). Corrected OD (cOD) differences were calculated based on differences in OD values for the antigen and control wells, the mean of positive and negative controls, and lot-specific parameters, as specified by the manufacturer. Equivocal results were re-tested and, if equivocal on retest, were categorized as positive for binary analyses.

**Appendix B. Detailed description of adjustments made to enzyme immunoassay results for variability in dried blood spots and validation against sera**

Three adjustments were made to the raw difference in OD values for each sample: an adjustment for differences in spot size; an adjustment for differences in elution volumes; and an adjustment for the diagnostic accuracy of DBS compared to sera.

**Differences in spot size**

The first adjustment was made to samples with blood spots less than 10 mm in diameter. To determine the adjustment, blood spots from the same positive control sample were spotted and left to dry overnight. Different spot sizes ranging from complete (10 mm) to 2 mm were cut in 2 mm increments and eluted according to the protocol in duplicate. The OD value of the antigen well was plotted against the spot size, and a linear regression was fit predicting the OD value of the antigen well for each unit increase in spot size. The following equation was used for the regression: $E\left( Y \right)=B0+B1(x)$; where Y= the OD value and x=the spot size. There was a mean 0.11 unit increase in OD value of the antigen well for each 1 mm unit increase in spot size. Results were the same for both measles and rubella assays. To adjust for samples of different spot size, a 0.11 unit increase in the OD value of the antigen well was added for each 1 mm decrease in spot size below 10 mm. Adjustments were made to 189 spots, of which 65 were 8 mm, 85 were 6 mm, 32 were 4 mm, 7 were less than 4 mm in circumference.

**Differences in elution volume**

The second adjustment was made to samples with eluted volumes less than 50 μl. A similar procedure was followed as that for the spot size adjustment, using the same positive control samples. Elution volumes were run in duplicate in decreasing volumes from 50 μl to 5 μl. The OD value of the antigen well was plotted against the spot size, and a linear regression was fit predicting the OD value of the antigen well for each unit increase in volume. The following equation was used for the regression: $E\left( Y \right)=B0+B1(x)$; where Y= the OD value and x=the eluate volume. There was a mean 0.03 unit increase in the OD value of the antigen well for each unit increase in volume, and this change was the same for both measles and rubella assays. To adjust for samples of different volumes, a 0.03 unit increase in the OD value of the antigen well was added for each 1 μl decrease in volume below 50 μl. One specimen had less than 50 μl.

**Accuracy of DBS**

The third adjustment was made to all samples to account for the use of DBS rather than sera for the assay kits. Paired sera and DBS samples from 203 individuals were tested for both measles and rubella IgG. The adjustment factor applied to the DBS samples was generated using a linear regression model. The outcome of the model was corrected difference in OD value (cOD) for the sera with the cOD value for the DBS used as the predictor. The cOD values for the DBS samples for this analysis were first adjusted for variations in spot size and eluate volume. The following equation was used for the regression: $E\left( Y \right)=B0+B1(x)$; where Y= cOD for sera and x=cOD for DBS. The association between the accuracy of the DBS cOD to predict the sera cOD differed between the measles and rubella kits. The model fits for measles and rubella IgG gave the following estimates for predicting the sera cOD respectively: $E\left( Y \right)=-0.17+0.47(x)$, $E\left( Y \right)=-0.49+0.87(x)$. These fits were used to adjust the DBS cOD values, which were then used to determine whether the samples were positive, negative, or equivocal and to estimate antibody concentration (IU/mL).

**Appendix C. Detailed description of prediction modeling to generate measles and rubella seroprevalence estimates for Southern Province, Zambia**

Generalized linear models with logit link were fit to rubella and measles serostatus (Table S2 and S3, respectively). Models considered individual and household level predictors that were chosen *a priori* given known characteristics of serostatus (i.e., age, vaccination status) or selected via random forest analyses. The variables assessed in the random forest analyses met two criteria: i) they were collected in clusters we wish to extrapolate to, and ii) they were completed by at least 50% of the sampled individuals. The predictors assessed were age, gender, maternal age, time to vaccination clinic, father’s education and occupation, religion, and whether an immunization card was available.

Figure S2 and S3 shows the results of the random forest analyses for rubella and measles, respectively. We found that immunization card (i.e., whether or not an immunization card was presented to the survey team) should be considered in the rubella predictive model (Figure S2). We did not consider MCV_routine (i.e., ever received routine measles vaccination) in the generalized linear models for rubella seroprevalence model due to the lack of variation in the variable across rubella seronegative individuals and resultant unrealistic standard error. For the random forest classification analysis for measles serostatus, we find that household level characteristics are important variables (i.e., father’s education, and occupation, maternal age, religion, and time to clinic) and were considered in the measles seroprevalence generalized linear model (Figure S3). It is important to note that both the measles and especially the rubella random forest models are over fit (Figure S4). In the random forest leave one out analysis, we found the area under the ROC curves greatly decreased. Ultimately, the lack of variation in our data (i.e., small number of seronegative samples: 6 rubella and 13 measles seronegative individuals among ages 9 months through 15 years old) meant measles and rubella serostatuses were not generalizable based on covariates.

The generalized linear models (Tables S2-S3) confirmed this finding. The leave-one-out analysis revealed that all models’ ability to correctly predict rubella serostatus greatly declined when removing just one observation (Table S2, area under the curve estimates < 0.63) (see Figure S4a for ROC curves). Models fit to measles serostatus showed that no household characteristic significantly improved the measles serostatus model, and that all models failed to accurately predict measles serostatus (Table S3, area under the curve estimates based on leave-one-out analysis < 0.50) (see Figure S4b for ROC curves). Rubella and measles serostatus was not generalizable based on covariates due to the small number of seronegative samples in this serological survey (6 rubella and 13 measles seronegative individuals among ages 9 months through 15 years old), and as a result seroprevalence could not be extrapolated to the Southern province.

**Table S2:** Logistic regression model results, the dependent variable is rubella serostatus (0=negative, 1=positive), N=305.

AIC = Akaike information criteria, AUC= area under the curve.

|  | **Model 1** | **Model 2** | **Model 3** | **Model 4** | **Model 5** |
| --- | --- | --- | --- | --- | --- |
|  | **OR (95% CI)** | **OR (95% CI)** | **OR (95% CI)** | **OR (95% CI)** | **OR (95% CI)** |
| Intercept | 49.66  (24.26,125.73) | 21.65  (6.13, 105.90) | 2.5  (0.54, 17.46) | 34.67  (13.07, 140.91) | 1.13  (0.13, 11.99) |
| Age |  | 1.16  (0.93, 1.53) |  |  | 24.33  (2.52, 227.24) |
| MR vaccination |  |  | 29.3  (3.51, 194.10) |  | 1.08  (0.83, 1.46) |
| Immunization card available |  |  |  | 1.87  (0.34, 10.23) | 2.44  (0.40, 16.06) |
| null log likelihood | -29.512 | -29.512 | -29.512 | -29.512 | -29.512 |
| log likelihood |  | -28.692 | -25.404 | -29.239 | -24.822 |
| log-like ratio test p-value |  | 0.2041 | 0.0042 | 0.4535 | 0.0248 |
| AIC | 61.024 | 61.371 | 54.7812 | 62.422 | 57.619 |
| AUC |  | 0.670 | 0.658 | 0.574 | 0.791 |
| Leave-One-Out Analysis AUC |  | 0.517 | 0.328 | 0.326 | 0.626 |

**Table S3.** Logistic regression model results, the dependent variable is measles serostatus (0=negative, 1=positive), N=282.

AIC = akaike information criteria, AUC= area under the curve. *Leave one out analysis AUC is not applicable for model 4 because there is not enough variation in measles serostatus and MCV routine vaccination status.

|  | **Model 1** | **Model 2** | | **Model 3** | **Model 4** | **Model 5** | **Model 6** | **Model 7** | **Model 8** | **Model 9** |
| --- | --- | --- | --- | --- | --- | --- | --- | --- | --- | --- |
|  | **OR (95% CI)** | **OR (95% CI)** | | **OR (95% CI)** | **OR (95% CI)** | **OR (95% CI)** | **OR (95% CI)** | **OR (95% CI)** | **OR (95% CI)** | **OR (95% CI)** |
| Intercept | 27.2 | 17.53 | | 13 | 10.8 | 17.07 | 9.56 | 20 | 10.96 | 29.63 |
|  | (15.30, 54.78) | (5.92, 64.71) | | (2.59, 236.18) | (1.94, 203.12) | (2.72, 346.62) | (1.68, 180.89) | (2.71, 441.98) | (1.50, 238.34) | (3.43, 1002.54) |
| Age |  | 1.07  (0.91, 1.29) | |  | 1.06  (0.89, 1.29) | 1.05  (0.88, 1.27) | 1.06  (0.89, 1.28) | 1.05  (0.89, 1.28) | 1.06  (0.89, 1.29) | 1.09  (0.91, 1.32) |
| MCV routine vaccination |  |  | | 2.21  (0.12, 13.17) | 1.80  (0.09, 11.98) | 1.61  (0.08, 11.54) | 1.52  (0.08, 10.27) | 1.71  (0.09, 11.76) | 1.82  (0.09, 12.31) | 2.41  (0.12, 17.24) |
| Father's Education |  |  | |  |  |  |  |  |  |  |
| *Ref: grade 1-12* |  |  | |  |  |  |  |  |  |  |
| other |  |  | |  |  | 0.20  (0.05, 0.99) |  |  |  |  |
| Father's Occupation |  |  | |  |  |  |  |  |  |  |
| *Ref: farm worker* |  |  | |  |  |  |  |  |  |  |
| other |  |  | |  |  |  | 4.22  (0.77, 78.54) |  |  |  |
| Religion |  |  | |  |  |  |  |  |  |  |
| *Ref: protestant* |  |  | |  |  |  |  |  |  |  |
| other |  |  | |  |  |  |  | 0.42  (0.09, 1.54) |  |  |
| Time to Clinic |  |  | |  |  |  |  |  |  |  |
| *Ref: <0.5 hour* |  |  | |  |  |  |  |  |  |  |
| > 1 hour |  |  | |  |  |  |  |  | 0.86  (0.16, 4.00) |  |
| 0.5-1 hour |  |  | |  |  |  |  |  | 1.08  (0.20, 6.03) |  |
| Maternal Age |  |  | |  |  |  |  |  |  |  |
| *Ref: <40 years old* |  |  | |  |  |  |  |  |  |  |
| 40+ years old |  |  | |  |  |  |  |  |  | 0.17  (0.01, 0.93) |
| null log likelihood | -43.214 | -43.214 | | -43.214 | -43.214 | -43.214 | -43.214 | -43.214 | -43.214 | -43.214 |
| log likelihood |  | -42.881 | | -42.993 | -42.762 | -40.81 | -41.466 | -41.911 | -42.715 | -40.651 |
| log-like ratio test p-value |  | 0.4119 | | 0.5068 | 0.6365 | 0.1864 | 0.3213 | 0.4565 | 0.91 | 0.1629 |
| AIC | 88.43 | 89.763 | | 89.987 | 91.524 | 89.619 | 90.932 | 91.822 | 95.429 | 89.303 |
| AUC |  | 0.583 | | 0.526 | 0.576 | 0.626 | 0.647 | 0.64 | 0.56 | 0.683 |
| Leave-One-Out Analysis AUC | | | 0.4 | NA* | 0.29 | 0.296 | 0.396 | 0.317 | 0.085 | 0.461 |

**Appendix D. Additional tables and figures**

**Table S1.** Measles vaccination coverage from campaign and routine immunization system, by age group

|  | Children ages 9 months - < 5 years (n=124) | | | | Children ages 5 - <16 years (n=244) | | | |
| --- | --- | --- | --- | --- | --- | --- | --- | --- |
|  | Vaccinated prior to survey according to:  % (95% CI) | | | | Vaccinated prior to survey according to:  % (95% CI) | | | |
|  | Vaccination card | Caregiver recall | Either  %^2^ | Missing | Vaccination card | Caregiver recall | Either  %^2^ | Missing |
| MR campaign  (ever vaccinated for rubella) | 39.6  (28.7, 51.5) | 51.5  (40.4, 62.6) | 91.1  (83.1, 95.5) | 1.1  (0.2, 5.5) | 39.2  (28.8, 50.6) | 50.1  (39.8, 60.4) | 89.3  (84.1, 92.9) | 9.3  (5.9, 14.4) |
| MCV1 | 32.9  (23.3, 44.2) | 51.5  (39.8, 63.0) | 83.4  (73.8, 89.9) | 2.7  (0.8, 8.3) | 7.6  (4.3, 13.0) | 79.8  (72.6, 85.4) | 87.4  (82.0, 91.3) | 10.3  (6.8, 15.5) |
| MCV2 (if >18 mo. of age)^1^ | 14.8  (8.8, 23.9) | 41.7  (30.4, 53.9) | 57.7  (45.4, 69.1) | 11.0  (5.6, 20.5) | 4.7  (2.1, 10.2) | 52.8  (42.0, 63.4) | 57.5  (46.8, 67.6) | 41.6  (31.5, 52.4) |
| Ever vaccinated for measles | 54.8  (43.4, 65.7) | 42.0  (31.6, 53.2) | 96.8  (91.2, 98.9) | 1.1  (0.2, 5.5) | 42.4  (32.2, 53.3) | 46.8  (37.0, 57.0) | 89.3  (84.1, 92.9) | 9.3  (5.9, 14.4) |

Note: Estimates account for survey weighting. 95% logit confidence intervals.

^1^ 103 children were ages 18-59 months and eligible for MCV2.

^2^ Vaccination card and caregiver recall do not sum to 100% due to weighting and rounding.

**Figure S1. Map of study clusters in PCES and serosurvey in Southern Province, Zambia**

*
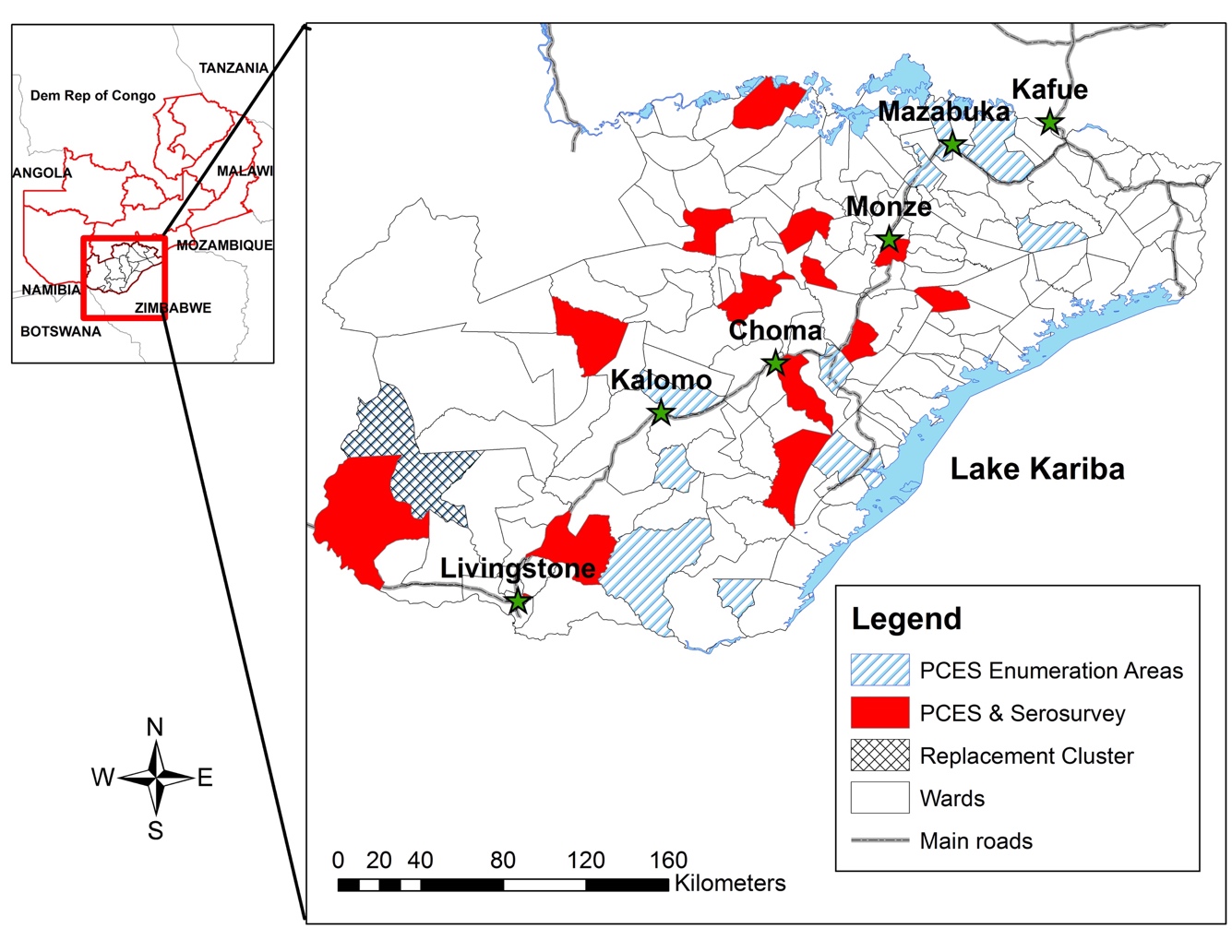
*

**Figure S2.** Variable importance plots resulting from a random forest classification of rubella serostatus with 10,000 trees.

**Figure S3.** Variable importance plots resulting from a random forest classification of measles serostatus with 10,000 trees.

**Figure S4.** Receiver Operating Characteristic (ROC) curves for rubella models (a) and measles models (b)

**Sources**

1. Mercader S, Featherstone D, Bellini WJ. Comparison of available methods to elute serum from dried blood spot samples for measles serology. *Journal of virological methods* 2006; **137**(1): 140-9.
